# Targeting Myelogenous Leukemia Stem Cells: Role of the Circulation

**DOI:** 10.3389/fonc.2012.00086

**Published:** 2012-08-02

**Authors:** Jane Liesveld

**Affiliations:** Hematology/Oncology Division, University of Rochester,Rochester, NY, USA

**Keywords:** leukemia stem cells, antibody, signal transduction, vascular interactions, targeting

## Abstract

Unlike stem cells from solid tumors, the stem cells which initiate myelogenous leukemias arise in marrow, an organ with a unique circulation which allows ready access of leukemia cells, including leukemia stem cells (LSCs), to the vasculature. This poses unique problems in the targeting of LSCs since these cells are found circulating in the majority of leukemia cases at diagnosis and are usually not detectable during remission states. Because most cases of leukemia relapse, it is suggested that LSCs remain quiescent in the marrow until they eventually proliferate and circulate again. This indicates that effective targeting of LSCs must occur not only in peripheral circulation but in the micro-circulation of the marrow. Targeting such interactions may overcome cell adhesion-mediated treatment resistance, other multi-drug resistance mechanisms, and opportunities for clonal evolution in the marrow environment. Targeting selectins and integrins, signal transduction mediators, and chemokine/cytokine networks in the marrow micro-circulation may aid in abrogating leukemia-initiating stem cells which contribute to disease relapse. LSCs possess surface antigen profiles and signal transduction activation profiles which may allow differential targeting as compared with normal hematopoietic stem cells.

## DEFINING THE LEUKEMIA STEM CELL AND ITS IMPORTANCE

Myeloid leukemias are a group of disorders arising in marrow and often involving blood which are characterized by overproduction of immature leukocytes or terminally differentiated cells as in chronic myelogenous leukemia (CML). In this review, we will focus on acute leukemias where impairment of differentiation is found. Most therapies available to treat acute myelogenous leukemia target leukemia blast cells which are in general a later progenitor/precursor cell no longer capable of self-renewal and multipotential differentiation. Only about 0.01% of normal marrow cells are hematopoietic stem cells (HSCs), and what the actual leukemia stem cell (LSC) number is at a typical new diagnosis of acute myeloid leukemia (AML) is not known. Also, it is not known if mutations which are associated with AML arise in normal stem cells or in more differentiated cell types which then acquire stem-like features (Misaghian et al., 2009). Leukemia stem cells are defined functionally as cells which are able to engraft in immunocompromised mice, but it is possible that xenotransplantation underestimates the numbers and types of stem cells due to the presence of a foreign microenvironment (Adams and Strasser, 2008). The concept that a LSC which is a counterpart to a normal HSC exists has been considered for many decades, but thus far the implications that such a cell has in the manifestation and therapy of acute myelogenous leukemia have remained controversial.

The cellular origin of AML remains unclear, with ongoing controversy as to whether it arises from a transformed true HSC with multipotential self-renewal capacity or in a more mature progenitor cell. Most AML cells do not possess the properties of self-renewal and multipotency. This has led to the concept of stem cell heterogeneity (Nguyen et al., 2012; Walter et al., 2012). Some leukemias may arise at the level of pluripotent CD33^-^ precursors, some may have an initial mutation in a pluripotent HSC with a collaborating mutation such as a core binding factor mutation occurring later, or some may arise at the level of a committed myeloid precursor as is the case in acute promyelocytic leukemia. Both the mutation(s) leading to a leukemic state and the cell of origin of the mutation are thought to have prognostic bearing (Walter et al., 2012). The LSC may not always be a rare population of quiescent cells. The phenotype, frequency, and functional properties of these cells may also change during disease progression (Mather, 2012; Nguyen et al., 2012).

Bonnet and Dick (1997) found that only leukemic blasts with the immature cell-surface phenotype characterized by CD34 expression with lack of CD38 expression were capable of transferring AML to immunodeficient mice; severe combined immunodeficiency (SCID) mice. This seemed consistent with a cancer stem cell (CSC) hypothesis which suggests that tumors are maintained by a small population of stem cell-like cancer cells which have the capability for indefinite self-renewal (Lapidot et al., 1994). Subsequently, some leukemia-initiating cells (L-ICs) have been found in the CD34 negative marrow fractions (Taussig et al., 2010). Also, LSCs have a hierarchy defined by length of repopulating potential in serial immune deficient mice transplantations defined as short- and long-term potential (Sarry et al., 2011). Those with long-term potential are thought to be quiescent (outside of active cell cycle) and express high levels of the multi-drug resistance phenotype, MDR-1, indicating ability to efflux cytotoxic drugs (Krause and van Etten, 2007). There is also evidence that pathways involved in proliferation of primitive cells such as Hox, hedgehog, notch, and Wnt are all involved in the maintenance and proliferation of the LSC (reviewed in Krause and van Etten, 2007).

While the CD34^+^CD38^-^ cells are clonal as based on cytogenetics, fluorescence *in situ* hybridization (FISH), and reverse transcriptase polymerase chain reaction (RT-PCR) in some cases, normal CD34^+^CD38^-^ cells are also capable of engrafting NOD/SCID mice and must be distinguished from their leukemic counterparts in the course of functional assays. If a multipotential CD34^+^CD38^-^ stem cell is the cell of origin for acute leukemia, it is not known why the lymphoid phenotype is suppressed after transformation. Satoh and Ogata (2006) have postulated that myeloid HSCs with minimal lymphopoietic potential may be the site of transformation in AML and could be a target to eliminate the LSC in many cases.

The LSC is best defined functionally by its ability to recapitulate leukemia faithfully in immunocompromised mice. This requires not only homing and engraftment potential to the murine microenvironment but ability to express the phenotype of the original AML in terms of surface phenotype and of clonal markers such as chromosome translocations or deletions or of other abnormal molecular markers such as nucleophosmin-1, Flt3–ITD expression, or Ras mutations. Unfortunately, only about 50% of AML cases have clonal chromosome markers to allow easy distinction, but other aberrant leukemic cell phenotypes can sometimes allow the distinction of normal vs. leukemia human CD45^+^ cells to be made by flow cytometry or by mutation analysis by PCR or sequencing. Detecting the presence of human CD45^+^ cells is not sufficient as normal HSCs are also able to engraft immunodeficient mice, so documentation of the leukemic nature of the engrafting cells is required. *In vivo* L-IC and HSC assays are important to measure functional stem cell capability and to measure effectiveness of therapies against L-ICs as has been determined with a compound kinetin riboside which has potential therapeutic efficacy and preferential effects against LSCs as compared with normal HSCs (McDermott et al., 2012). Some AML do not engraft immune deficient mice, and it is thought that murine engraftment could represent proliferative potential of the leukemic cells or could simply reflect ability to interact with the murine microenvironment (Risueno et al., 2011). Targeting LSCs is thought to be of importance since the burden of LSCs at diagnosis has prognostic significance. Patients whose blasts at diagnosis fail to engraft NOD/SCID mice at high cell doses have superior long-term survival (Pearce et al., 2006).

Knowing which “stem cell” to target therapeutically in AML is difficult, however, since relapse may occur in a founder clone, a recurring subclone, or in a novel stem cell clone (Walter et al., 2012). Not only do controversies exist about how to identify a LSC but also about whether such a stem cell must be eliminated in order to effectively treat the leukemia (Kelly et al., 2007; Majeti, 2011). The possibility that stem cell-like components of tumors may change phenotype rapidly and reversibly also makes study of these cells difficult (Mather, 2012). Because of the heterogeneity in the phenotype of LSCs, surface antigen phenotype is inadequate as a means of isolation. High expression of aldehyde dehydrogenase (ALDH) activity in conjunction with CD34 has been found to delineate an L-IC (Ran et al., 2012). The frequency of aldehyde bright cells in the marrow at time of initial diagnosis is an independent prognostic factor predicting overall survival (Ran et al., 2012). It has also been shown that in a majority of AML cases, two subsets with progenitor immunophenotype coexist, and both have LSC activity and are hierarchically patterned (Goarden et al., 2011).

That the stem cell model has clinical significance in AML is suggested by studies such as one which showed that the percentage of CD34^+^CD38^-^ LSCs at the time of diagnosis correlated with the duration of relapse-free survival in 92 patients (van Rhenen et al., 2005). Those with >3.5% AML LSCs had a median relapse-free survival of 5.6 months vs. those with <3.5% who had a median relapse-free survival of 16 months. The presence of a CD34^+^CD38^-^ ALDH intermediate population has been shown to correlate with minimal residual disease (MRD) and subsequent relapse and to be able to distinguish LSCs from normal stem cells which are aldehyde high expressors (Gerber et al., 2012). Overall, LSC frequency is thought to be an important component of MRD (Buccisano et al., 2012).

## ASSAYING THE LSC

The LSC can be assayed by flow cytometry and immunophenotype, but given the vagaries and lack of standardization of this technique, it is most accurately assayed by its ability to engraft immune deficient mice, although as noted above, there are both quantitative and qualitative problems with those assays (Mather, 2012). The L-IC is able to recapitulate the phenotype of the original leukemia when transplanted into NOD/SCID mice and then to be secondarily transplanted to another sublethally irradiated mouse. The NOD/SCID xenograft has no B or T cells, and leukemia engraftment is often quite low and requires transplantation of thousands of cells. In the NOD/SCID/IL-2Rγ-/- (NSG) species, NK cells are also deficient and as few as 100 cells will transplant the leukemia (Ishikawa et al., 2005). ALDH has been utilized as a marker of LSCs, and *in vitro* long-term culture assays have been reported to identify an L-IC (Sutherland et al., 2001).

Initially, it was thought that the LSC resided in the CD34^+^CD38^-^ compartment, but many anti-CD38 antibodies, including those utilized in the original isolation of LSCs inhibit engraftment of CD34^+^CD38^+^ leukemia cells through an Fc-dependent mechanism (Taussig et al., 2008). Blockade of Fc receptors can result in engraftment of CD38^+^ AML cells, suggesting that CD38^+^ fractions may contain LSCs (Taussig et al., 2008). Also, in some AML cases with nucleophosmin mutation, the L-ICs reside in the CD34 negative fraction (Taussig et al., 2010).**Table1** illustrates some of the characteristics of AML stem cells as contrasted with normal HSCs.

**Table 1:** Sample characteristics of leukemia vs. normal stem cells.

	LSC	HSC
Frequency	Usually rare	Rare
Cell cycle status	Quiescent	Quiescent
MDR-1 expression	+	+
CD34 expression	+	+
CD38 expression	Sometimes	-
HLA-DR expression	-	-
CD71 expression	-	-
CD33 expression	-(Usually)	-
CD117 expression	-	+
CD90 expression	-	+
CD123 expression	+	-(Usually)
Aldehyde dehydrogenase expression	+ But intermediate	+ (Bright)

*LSC, leukemia stem cell; HSC, hematopoietic stem cell; MDR, multi-drug resistance.*

## MEANS TO TARGET LSCs

### TARGETING CELL-SURFACE MOLECULES ON LSCs

One means of isolating and potentially targeting LSCs would be to target cell-surface molecules on the cell surface which are selectively or differentially expressed relative to normal tissue, in this case the normal HSC. Such antibodies could be expressed on differentiated normal cells but not on normal HSCs. LSCs lack Thy-1 (CD90) and c-kit (CD117), both found on normal HSCs, but they possess CD123, CD33, and C-type lectin-like molecule-1 (CLL-1). CD96, CD45, CD32, and CD25 have been shown to be preferentially expressed on AML stem cells as compared with normal HSCs (reviewed in Majeti et al., 2009). CD90 is expressed by normal but not AML stem cells (Baum et al., 1992). LSCs do not express human leukocyte antigen (HLA)-DR or CD71 (Blair et al., 1998). Many of these antigens are expressed on the cell surface, allowing stem cell targeting throughout the circulation without specific delivery requirements.

### ANTIGENS USED IN TARGETING AML LSCs

#### CD33

CD33 is a sialylated protein expressed on normal cells with myelomonocytic differentiation, AML blasts, and some LSC populations. Numerous antibodies to CD33 have been developed for clinical use, including gemtuzumab ozogamicin, a recombinant humanized anti-CD33 conjugated with calicheamicin (Hamann et al., 2002). This conjugate induced remission in relapsed AML, most cases being CD33^+^, but it also had some activity in CD33^-^ cases probably due in large part to the calicheamicin effects (Jawad et al., 2010; Pollard et al., 2012). It was also able to eliminate promyelocytes in cases of acute promyelocytic leukemia. While not studied directly, it was thought to differentially kill leukemia cells and possibly LSCs, but prolonged cytopenias also suggested in some cases that it was expressed on normal HSCs as well (Walter et al., 2012). Gemtuzumab ozogamicin (anti-CD33 conjugated with calicheamicin) was approved for the treatment of relapsed AML in elderly patients. Because CD33 is also expressed on endothelial cells of certain vascular beds, toxicities such as sinusoidal obstructive syndrome of liver occurred in some cases (McDonald, 2002), leading to eventual voluntary withdrawal of this conjugated antibody from the market. It is still being utilized in clinical trial settings, however, so future studies may be able to discern its effects on LSCs in states of MRD and other therapeutic settings in leukemia (Castaigne et al., 2012). Other antibody conjugates are also under development (Walter et al., 2012).

CD33 is a relatively hard molecule to target due to relatively low antigen expression (about 104 CD33 molecules/cell) and slow conjugate internalization (Walter et al., 2012). In CML, it has been found (Hermann et al., 2012b) that CD34^+^CD38^-^CD123^+^ cells expressed significantly higher levels of CD33 compared to normal CD34^+^CD38^-^ stem cells. Levels of CD33 mRNA were also higher. Gemtuzumab ozogamicin inhibited these in long-term culture initiating cell assays, but no mention was made of inhibition of engraftment in NOD/SCID mice, however.

#### CD123

CD123 is the high-affinity interleukin-3 receptor alpha chain. While it is differentially expressed on leukemia vs. normal stem cells (Jordan et al., 2000), cord blood may express CD123 positive progenitors. CD123 has been targeted for clinical use with a diphtheria toxin–IL-3 fusion protein (Kuo et al., 2009) and numerous additional means to target CD123 have been developed. Jin et al. (2009) reported on a monoclonal antibody, 7G3 which targets CD123. It was able to reduce AML-LSC engraftment and improve murine survival in models of pre-established leukemia where the antibody showed reduced AML burden in the marrow and periphery as well as impaired secondary transplantation after 7G3 exposure, indicating that LSCs had been inhibited. Stein et al. (2009) used single chain Fv antibody fragments specific for CD123 designed to specifically target LSCs. This binding created two cell death inducing molecules; one being immunotoxic through truncation with a pseudomonas exotoxin and the other targeted to the low-affinity Fcγ receptor III (CD16). This construct was able to generate potent lysis of MOLM-143, and SKNO-1 cells in ADCC reactions. None of the CD123 targeted agents have had significant clinical development to date.

#### C-type lectin-like molecule 1

This molecule has been found to be expressed on CD34^+^CD38^-^ AML cells. Its expression tracked with clinical remission or relapse. It was not found on CD34^+^CD38^-^ cells from normal marrow or on cells regenerating post-chemotherapy (van Rhenen et al., 2007). Zhang et al. (2011) have developed nanoparticles covalently decorated with CLL-1 targeting peptides. These are loaded with daunorubicin which is then internalized to LSCs but not to normal HSCs. Other antibody means of targeting CLL-1 are also under development (Zhao et al., 2010).

#### CD96

CD96 is a member of the immune globulin superfamily. It is normally found on T-lymphocytes. It is thought to be LSC-specific as it is found on most CD34^+^CD38^-^ AML cells whereas it is only expressed weakly in the normal counterparts (Hosen et al., 2007). When leukemic cells were separated into CD96 positive and negative cells, only positive cells engrafted Rag-deficient mice, suggesting that this may be an LSC-specific therapeutic target.

#### Anti-PR1/HLA-A2 T-cell receptor

Antibodies against PR1 induce complement-dependent cytotoxicity against AML progenitor cells. PR1 is a HLA-A2 restricted leukemia-associated peptide from proteinase 3 (P3) and neutrophil elastase (NE) recognized by anti-PR1-specific cytotoxic T lymphocytes which can contribute to cytogenetic remission of AML. These cells cause specific lysis of AML, including LSCs (Sergeeva et al., 2011).

#### CD44

The CD44-6v isoform is differentially expressed by AML progenitor cells. An activating CD44-specific antibody was able to eradicate AML but not normal cord blood and normal adult marrow engraftment in NOD/SCID mice (Jin et al., 2006). *In vivo*, the effect of this antibody, H9 was limited to situations with a lower burden of disease (Jin et al., 2006).

#### CD32

This antigen has been reported by Saito et al. (2010) to be differentially expressed on the AML stem cell. It recognizes the FcRIIa low-affinity receptor and as such is expressed on monocytes and other effectors of ADCC as well.

#### CD25

Saito et al. (2010) also described this on the AML stem cell. Either CD32 or CD25 or both were expressed in 32/61 (53%) of patients in AML LSCs which were able to initiate AML and were cell–cycle quiescent and chemotherapy resistant *in vivo*.

#### CD47

CD47 is a ligand to SIRP-alpha (signal regulatory protein-alpha). Its expression sends a “don’t eat me” message to phagocytic cells. Blocking antibodies to CD47 enabled phagocytosis of human AML LSCs but not normal LSCs (Jaiswal et al., 2009). This antibody has been found effective in a treatment model where NSG mice were transplanted with LSCs. After 8–12 weeks the mice were treated with control IgG or blocking antibodies to CD47. The antibodies to CD47 resulted in rapid clearance of AML in blood and also of marrow LSCs. There was absence of leukemia in secondary recipients (Majeti et al., 2009). CD47 is expressed on many tissues, and it may be more effective with chemotherapy or with mobilizing agents to facilitate phagocytosis in the peripheral circulation (Majeti et al., 2009).

### SUMMARY OF ANTIBODY TARGETING

While antibodies have been developed to target differentially expressed surface markers on AML vs. normal cells, none of these have had significant impact on disease outcomes to date. In part, this may be due to lack of complete specificity, inadequate targeting, toxicities due to non-specificity, antigen modulation, and subsequent resistance, or other as yet undiscovered mechanisms. Despite its removal from the market in the United States, the anti-CD33 antibody with which most experience developed, gemtuzumab ozogamicin, has recently been tested in low doses during induction and consolidation chemotherapy for AML and has been found to result in superior overall and disease-free survival when used in combination with chemotherapy (Castaigne et al., 2012). LSC frequency or MRD was not measured as part of the study, so whether this relates to stem cell targeting or other mechanisms of leukemia suppression remains uncertain.

### TARGETING SIGNALING PATHWAYS IN LSCs WITH SMALL MOLECULE INHIBITORS

One approach to eliminating the LSC would be to target pathways regulating stem cell self-renewal, particularly if these are differentially expressed in LSCs vs. HSCs (Rasheed et al., 2011). For example, inhibitors of Wnt have been examined in CML (Baghdadi et al., 2012) and notch inhibitors have been examined in ALL, but these are also toxic to normal HSCs in some cases, indicating a need to find selective inhibitors or relatively selective inhibitors for LSCs (Guzman and Jordan, 2004; Mikkola et al., 2010). Heat shock protein inhibitors have been found to be inhibitory to AML progenitor cells, but whether those represent true stem cells is uncertain (Hermann et al., 2012a).

#### PI3K/AKT/mTOR PATHWAY

The PI3K/AKT/mTOR pathway is activated in the majority of AML cases, but the effects of inhibiting this pathway on LSC frequency have not been described in detail (Mikkola et al., 2010). In mice, when PTEN, normally a suppressor of the PI3K/AKT/mTOR pathway is depleted, normal HSCs are depleted, but L-ICs are expanded (Lee et al., 2010). The mTOR inhibitor, rapamycin, blocks this. The depletion of Pten-deficient HSCs was not caused by oxidative stress (Peng et al., 2010).

Altman et al. (2011) have found that dual targeting of mTORC1 and mTORC2 results in potent suppressive effects on primitive leukemic progenitors from AML patients. Inhibition of the mTOR catalytic site with OSI-027, a dual mTORC1/mTORC2 inhibitor, resulted in suppression of mTORC2 and mTORC1 complexes and elicited a much more potent anti-leukemic response than selective mTORC1 targeting with rapamycin. Whether these inhibitors act at a true stem cell level remains unclear.

#### NF-κB pathway

NF-κB has been found to be active in most AML stem cells but not in normal lineage negative progenitors (Jordan et al., 2006). The proteasome inhibitor, MG-132, inhibited NF-κB activation through stabilization of its cellular inhibitor, IκB and was able to induce apoptosis in CD34^+^CD38^-^ AML cells while sparing normal progenitors. Since that report, other inhibitors of NF-κB such as bortezomib or specific inhibitors of IκB kinase which phosphorylate and inactivate IκB have been developed and have been reported to be inhibitory to AML progenitor cells (Frelin et al., 2005). The drug parthenolide has IKB-Iκκ inhibitory activity and is able to induce apoptosis in AML cells coincident with NF-κB inhibition, p53 activation, and induction of reactive oxygen species (Neelakantan et al., 2009).

#### Tyrosine kinase inhibitors

These have been utilized primarily to target bcr/abl, the driving mechanism in CML. While most bcr/abl inhibitors such as imatinib mesylate, nilotinib, and dasatinib eradicate most CML cells, they are ineffective against a reservoir of quiescent LSCs (Baghdadi et al., 2012). Inhibition of the Wnt/beta-catenin, hedgehog, and histone deacetylases has been shown to have potential to inhibit the CML stem cell.

#### Hedgehog/smoothened pathway

Hedgehog signaling is essential for maintenance of CSCs (Zhao et al., 2009). Loss of smoothened, an essential component of the Hedgehog pathway, impairs HSC renewal and decreases induction of CML by bcr/abl. Inhibition of hedgehog by cyclopamine inhibited the propagation of CML. In contrast, hedgehog signaling is not required for adult murine HSC function and hematopoiesis (Su et al., 2012). Its role in an AML LSC is not well worked out yet, but inhibitors of the Wnt and Shh signaling pathway cause less apoptosis in normal vs. leukemic HSCs (Hofmann et al., 2009).

#### Oxidative stress pathways

While states of low oxidative stress are thought to aid in maintenance of stem cell quiescence, CSCs may be susceptible to agents which induce oxidative stress. In fact, excessive production of ROS or a deficiency in antioxidant pathways has been noted in acute leukemias (Hole et al., 2011). This has not been studied systematically as a means of differential LSC targeting.

### TARGETING VASCULAR INTERACTIONS

Since LSCs as well as their blast progeny can be isolated from the vasculature in which they circulate, it is likely that exploitation of LSC–vascular interactions would have greater impact on targeting these stem cells than would be the case in other cancers where the stem cells do not routinely circulate except at times of active metastasizing. Furthermore, the LSC arises in the marrow microenvironment, an organ that is very vascular, again suggesting a potential role for targeting leukemia/vascular interactions. In the marrow, LSCs interact with cells, cell matrix, and soluble factors in the microenvironmental niche (Konopleva and Jordan, 2011). In mice, the microenvironmental niche has been characterized as vascular and osteoblastic, but whether such distinct niches exist in human marrow is uncertain.When leukemia cells enter the blood, they must traverse the endothelial cells lining the marrow sinusoids which comprise the vascular niche, and when they return to the marrow, they are engaged by selectins for rolling on the endothelial layer after which they attach via integrins or other adhesins. Thereafter, they transmigrate the endothelial barrier before again lodging in the marrow niche.

#### Role of endothelial cells

As noted above, when LSCs and other AML cells home to marrow or egress marrow to the peripheral circulation, they must traverse an endothelial monolayer. These form an intact lining of marrow sinusoidal vessels. The homing process involves rolling on selectin surfaces, engaging integrin or other adhesion receptors, transmigrating the endothelial layer, and lodging in the marrow hematopoietic niche. Chemotherapy resistant AML stem cells are thought to engraft with the marrow endosteal region (Ishikawa et al., 2004). Stem cell interactions with the niche can therefore theoretically be disrupted at each of these steps with possible impact on survival, proliferation, and function.

Sipkins et al. (2005), using intravital microscopy showed that NALM-6, an ALL cell line, engrafted in microvascular domains in the marrow. Colmone et al. (2008) demonstrated that the NALM-6 cells actively disrupted normal vascular niches for transplanted human CD34^+^ cells and caused normal CD34^+^ cells to leave their niches and to engraft in alternative niches. Targeting the supportive function of the vascular niche in marrow and circulation may therefore have inhibitory activity against LSCs. Whether this will have a differential effect as compared with normal stem cells remains uncertain.

Targeting vascular endothelial growth factor (VEGF) and its receptor, VEGFR2 (KDR) may inhibit both AML cells and endothelial cell proliferation as both possess these receptors (Ziegler et al., 1999). Marrow vascularity is increased in AML, and targeting this vasculature may decrease AML proliferation. The role of specific angiopoietins such as basic fibroblast growth factor or transforming growth factor-beta has not been examined as related to AML stem cell survival, but this might also represent pathways which might be targeted to decrease proliferation of AML cells (Folkman, 2001). It has also been demonstrated that leukemia cells secrete factors which can enhance endothelial cell proliferation which in turn can stimulate leukemia cell proliferation and survival (Veiga et al., 2006). Targeting endothelial cells via inhibition of the angiopoietin–Tie2 axis (Schliemann et al., 2007) or via combretastatins (Petit et al., 2008) might also inhibit leukemia cell proliferation, but specificity for LSCs has not been determined.

#### CXCL12 and CXCR4

The CXCL12–CXCR4 chemokine–chemokine receptor axis is involved in AML stem cell interaction with the marrow as is also the case with normal HSCs. CXCR4 is expressed on primary leukemic cells, and high expression is a negative prognostic factor (Rombouts et al., 2004). Treatment with the CXCR4 antagonist, plerixafor, has been found to increase sensitivity of Flt-3 mutated AML cells to the FLT3 inhibitor, sorafenib and to induce the mobilization of AML cells into the peripheral blood (Zeng et al., 2009). It is uncertain whether there is preferential mobilization of LSCs vs. normal progenitors and bulk AML cells.

#### Anti-VLA-4 and anti-VCAM

The integrin–integrin receptor pair of very late antigen-4 and vascular cell adhesion molecule-1 is involved in retention of AML cells in marrow. AML blasts with higher CD49d expression have a poorer prognosis (Matsunaga et al., 2003), but the function of very late-antigen-4 as measured by binding of soluble VCAM-1 may be associated with improved overall survival of patients (Becker et al., 2009). Interactions between VLA-4 and fibronectin or VCAM may modulate chemotherapy response. How this impacts AML stem cells remains uncertain. Targeting other integrin-linked pathways may also inhibit AML stem cells (Muranyi et al., 2010).

#### Role of CD44

CD44 is a glycoprotein which binds to hyaluronan, selectins, and osteopontin and has pleiotropic functions in organogenesis, cell homing, and migration (Krause et al., 2006). High levels of the molecule on AML cells are associated with higher relapse rates (Quere’ et al., 2011). The anti-CD44 antibody, H90 targets LSCs in human AML through niche disruption and with evidence of LSC differentiation and loss of LSC self-renewal capacity as compared with effects on normal HSCs. Jin et al. (2006) found that using an activating monoclonal antibody to CD44, leukemic repopulation of NOD/SCID mice was markedly reduced probably due to interference with transport to the stem cell-supportive niches. Since CD44 is ubiquitously expressed, it is not known if the differential effects on LSCs are due to alternative splicing or other reasons.

#### Role of hypoxia

The marrow microenvironment in AML is relatively hypoxic (Fiegl et al., 2009). Under normoxic conditions, it has been found (Wang et al., 2011) that HIF-1α signaling was selectively activated in the stem cells of murine lymphoma and human AML. HIF-1α shRNA and HIF-1α inhibitors abrogated the colony-forming unit (CFU activity) of human CSCs. The inhibitor echinomycin eradicated serially transplantable human AML cells in xenogeneic models by preferential elimination of leukemic-/cancer-specific stem cells. The increased HIF-1 activity in cancer stem cells was seen in both hypoxic and normoxic states. Rakicidin A is another compound which may target a hypoxic microenvironment (Yamazaki et al., 2007).

#### Role of selectins

Leukemic stem cells express sialylated carbohydrate ligands on their surfaces that adhere to selectin proteins found on endothelial cells. In marrow vascular beds, these selectins are constitutively expressed. Immobilized selectin protein can be utilized as a targeting mechanism for cells under flow and can capture some LSCs. Mitchell et al. (2012) have utilized targeted liposomal doxorubicin (L-DXR) functionalized with recombinant human E-selectin and polyethylene glycol. Normal leukocytes exposed to these molecules demonstrated minimal cell death, whereas leukemia cells could be targeted. This mechanism was effective either in suspension or immobilized onto microtubular devices. P-selectin coated microtubules have been found to enrich for some hematopoietic progenitors from marrow through differential rolling (Narasipura et al., 2008), and this observation may allow delivery of cytotoxics to cancer cells (Rana et al., 2009), but whether this will have a differential effect on LSCs vs. other leukemia progenitors is still unclear.

### ROLE OF CELL CYCLE IN THE VASCULAR NICHE

Chemotherapy agents used to treat leukemia kill cells that are in active cell cycle but spare LSCs which are in a quiescent state in the marrow endosteal niche. Like normal stem cells, LSCs are predominately in a quiescent state which allows avoidance of stem cell exhaustion and minimization of risk of oncogenic events. Those LSCs located more centrally in the marrow cavity have a higher likelihood of being in active cell cycle (Doan and Chute, 2012). Attempts have been made to use agents such as granulocyte colony stimulating factor to cause LSCs to cycle, disrupting them from their vascular niche and increasing their sensitivity to agents such as cytarabine(Mikkola et al., 2010). Such efforts have not had great therapeutic impacts, however (Pabst et al., 2012).

## SUMMARY

The LSC, like its normal stem cell counterpart, lodges in marrow niches and occasionally circulates in the blood. Ideally, a therapeutic strategy that targets LSCs must spare self-renewing normal HSCs so as to protect ongoing hematopoiesis. The comparison of LSCs vs. HSCs functionally is crucial to identifying unique properties of the LSC which can be targeted. These will include surface antigen phenotyping (Amadori and Stasi, 2006), comparison of signal transduction pathway activation, response to reactive oxygen species and stress protein responses at baseline and in response to chemotherapeutic agents. Also, understanding differences in interactions with vasculature peripherally and in marrow may allow for differential targeting of LSCs (Doan and Chute, 2012). The ideal target will thus be expressed on a large fraction of LSCs, be stable through treatment types and disease stages, be present on cell-cycle quiescent AML-initiating cells residing within the endosteal niche, and be non-toxic to normal HSCs. Determining the clinical effectiveness of agents which target stem cells and proving that this is clinically relevant is hard to achieve even in AML, but novel agents and appropriate biomarkers may allow application of these strategies for the future (Rasheed et al., 2011). LSCs are heterogeneous, grow in subclones which have some degree of genetic instability, and evolve with time. Many LSC-derived subclones may be undetectable at diagnosis, but after therapy, they may become the dominant clone with relapsing disease (Nguyen et al., 2012). Both rare cancer stem cells and dominant clones will need to be eliminated for successful disease control (Adams and Strasser, 2008). Understanding the interaction between LSCs and microenvironmental components may also elucidate means to target these rare cells (Klyuchnikov and Kroger, 2009). Due to the heterogeneity of the LSC and the similarities between HSCs and LSCs, many challenges remain in effective targeting of these rare cells which are thought to require elimination if AML therapies are to have long-term success (Krause and van Etten, 2007).

## Conflict of Interest Statement

The author declares that the research was conducted in the absence of any commercial or financial relationships that could be construed as a potential conflict of interest.
